# 5-Fluorouracil combined with cisplatin *via* arterial induction for advanced T-stage nasopharyngeal carcinoma: A 10-year outcome of a phase I/II study

**DOI:** 10.3389/fonc.2022.868070

**Published:** 2022-07-27

**Authors:** Li Xiang, Yun Zheng, PeiRong Ren, Sheng Lin, JianWen Zhang, QingLian Wen, LiJia He, ChangLing Shang, JingBo Wu

**Affiliations:** Department of Oncology, Affiliated Hospital of Southwest Medical University, Luzhou, China

**Keywords:** cisplatin, fluorouracil, advanced T-stage NPC, temporal arteries, intra-arterial chemotherapy

## Abstract

**Background and Purpose:**

Currently, there is no optimal dose recommendation for a 120-h continuous infusion of 5-fluorouracil *via* arterial cannulation for advanced T-stage nasopharyngeal carcinoma (NPC). Thus, the aim of this study was to determine the maximum tolerated dose (MDT), along with the efficacy, late adverse events, and 10-year survival outcome of 5-fluorouracil administered continuously for 120 h combined with cisplatin *via* the superficial temporal artery in patients with advanced T-stage NPC.

**Materials and Methods:**

Fifty-one patients with histologically confirmed advanced T-stage NPC were eligible for inclusion in this clinical trial. The patients received induction chemotherapy consisting of cisplatin (20 mg/m^2^/d for 1–5 d) and 5-fluorouracil, administered continuously for 120 h at different dose gradients *via* a superficial temporal artery. To identify the MTD of 5-fluorouracil infused arterially, we employed a 3 + 3 design during study phase I. The initial dose administered was 200 mg/m^2^/d, which then was gradually escalated by 50 mg/m^2^/d until the MTD was reached. Following two cycles of induction chemotherapy, current radical chemoradiotherapy commenced. We assessed the efficacy, survival, toxicity, and quality of life of patients following treatment.

**Results:**

The overall response (complete response + partial response) rates following induction chemotherapy in the primary mass and lymph nodes were 100% and 100%, respectively. All 51 (100%) patients achieved T-category down-staging after intra-arterial chemotherapy. The MTD was 450 mg/m^2^/d for 120 h. No late neurological toxicities, such as brain stem injury, temporal lobe necrosis, and spinal cord injury, were observed. The 5- and 10-year overall survival (OS) rates were 78.0% and 71.7%, respectively, with a median OS of 131 months.

**Conclusion:**

Continuous infusion of 5-fluorouracil combined with cisplatin *via* the superficial temporal artery showed promising survival benefits and few toxicities in patients with advanced T-stage NPC.

## 1 Introduction

Owing to the insidious site of tumorigenesis and varying clinical symptoms, more than 70% of newly diagnosed nasopharyngeal carcinoma (NPC) cases are classified as locoregionally advanced tumours (1, 2). Low doses administered to avoid the irradiation of organs at risk (OAR) might not eliminate the tumour completely, leading to treatment failure of advanced stage NPC owing to recurrence. Intensity-modulated radiotherapy (IMRT) can achieve excellent 5-year local control (LC) rates of > 90% in patients with T1–T3 tumours. However, the treatment of T4 tumours is challenging as these tumours often lie close to critical neurological structures, compromising the radiation dose coverage area and, therefore, undermining LC (3). Induction chemotherapy (IC) has potential advantages, including decreasing the tumour volume and allowing dose constraints to avoid adjacent OAR, that are advantageous to patients with advanced T-stage NPC. The inclusion of concurrent cisplatin plus 5-fluorouracil (PF) to conventional-fractionated radiotherapy is recommended by the National Comprehensive Cancer Network for the treatment of locoregionally advanced NPC.

The induction regimen of a combination of cisplatin and continuous infusion of fluorouracil is administered intravenously. Systemic administration has limitations with respect to low local drug concentrations and high systemic venous drug levels. In a Phase III, multicentre, randomised controlled trial conducted to verify the efficacy of IC for locoregionally advanced NPC, 73% of patients with grade 3 or 4 tumours suffered adverse effects, and there was no increase in locoregional failure-free survival (4). In another study, IC led to T-category down-staging in only 35% of patients with advanced T-stage cancer; however, 50% of patients exhibited acute toxicity to IC (5). In a trial to assess the pharmacokinetics of 5-fluorouracil in the plasma and tumour tissue during 5-d continuous infusion therapy in patients with cancer, the 5-fluorouracil levels in plasma were 3–8-fold higher than those in the tumour tissue. The optimisation of cancer therapy by enhancing drug delivery is of major interest because of the identification of several barriers to drug delivery, underlining the need for novel therapeutic strategies to increase drug delivery to tumour sites (6).

Super-selective intra-arterial chemotherapy for NPC has the advantage of delivering high concentrations of chemotherapeutic drugs to the primary tumour with fewer systemic toxicities than systemic administration. The superficial temporal approach is technically simple and probably the easiest method to insert a catheter into the lingual, facial, or maxillary artery (7). In a preoperative therapy study, which used this approach with low dose radiotherapy for 30 patients with T3 or T4 squamous cell carcinoma of the head and neck, all 30 patients (100%) exhibited complete responses. Furthermore, the 5-year survival rate was 70.2%, and patients mainly present with local toxicity, with less systemic toxicity (8). Several randomised trials of long-term 5-fluorouracil infusion have demonstrated an improved therapeutic index with respect to efficacy and toxicity compared with short-term infusion (9).

Prolonging the 5-fluorouracil infusion from 96 to 120 h in patients with previously untreated stage IV head and neck cancer increases the complete response rate from 19% to 63% (10). To the best of our knowledge, there is no optimal dose recommendation for a 120-h continuous infusion of 5-fluorouracil *via* arterial cannulation for advanced T-stage NPC. Therefore, in this study, we aimed to determine the optimal dose and maximum tolerated dose (MTD) of 5-fluorouracil and its adverse effects following continuous pump infusion for 120 h *via* cannulation of the superficial temporal artery. The clinical significance and indications were also determined. The MTD and short-term efficacy, as well as the 10-year follow-up results, are reported here.

## 2 Materials and methods

### 2.1 Ethics approval and consent

This Phase I/II study was conducted in accordance with the guidelines of the Helsinki Declaration, and registered in the China Clinical Trial Center (ChiCTR-TRC-1900027372, http://www.chictr.org.cn/showproj.aspx?proj=29617). Written informed consent was obtained from all patients before participation.

### 2.2 Patients and pre-treatment evaluation

Between May 2004 and October 2010, newly diagnosed patients with non-metastatic, histologically confirmed T3 or T4 (classification by the sixth edition of the American Joint Committee on Cancer staging system) NPC were prospectively recruited. The inclusion criteria were as follows: patients with previously untreated NPC; adequate bone marrow, hepatic, heart, and renal action; absence of pregnancy or lactation; absence of previous malignancy or other concomitant malignant disease; and aged 18–70 years with an Eastern Cooperative Oncology Group performance status of 0 or 1. The essential pre-treatment assessments included the following: complete medical history taking and physical examination, haematology and biochemistry tests, nasopharyngeal fibreoptic endoscopy, enhanced chest computed tomography (CT), abdominal ultrasound/CT, enhanced magnetic resonance imaging or CT of the nasopharynx and neck, nasopharyngoscopy, and bone emission CT.

### 2.3 Superficial temporal artery catheterisation

Before chemotherapy, three-dimensional CT angiography of the carotid artery was performed to identify whether the superficial temporal artery was the main tumour-feeding artery. The entire operation was performed with patients under local anaesthesia of the temporal region by an experienced oral surgeon. After a guiding catheter was advanced into the superficial temporal artery, an indwelling vascular catheter was retrogradely inserted into the main feeding artery. During the operation, methylene blue staining was used to differentiate the skin and mucous membranes from the tumour area, as well as to adjust the depth of the tube and to reach the ideal position. The flow in the target artery was checked by CT angiography after infusion of the contrast agent. If the nasopharyngeal tumour exceeded the midline, bilateral superficial temporal artery cannulas were inserted. Furthermore, pre-treatment confirmation of the feeding artery was performed by injecting small amounts of methylene blue. Local heparinization was performed during catheterisation.

### 2.4 Superficial temporal artery chemotherapy and trial design

All anticancer agents were injected using a superficial temporal arterial catheter. Dose-limiting toxicity (DLT) was defined as grade III or above oral mucositis or diarrhoea and grade IV or above other serious toxicities, which were not ameliorated even after 1 week of symptomatic treatment. The Phase I study protocol employed a 3 + 3 design. In this design, three patients were treated with dose K. If none of the patients experienced DLT, the dose was escalated to K+1. If two or more patients experienced DLT, the dose was de-escalated to K-1. If one patient experienced DLT, three more patients received dose K. If one of the six patients experienced DLT, the dose was escalated to K+1. If two or more of the six patients experienced DLT, the dose was reduced to K-1 and the trial was closed. The dose K-1 was considered to be the maximum tolerated dose. To determine the MTD of 5-fluorouracil, we used 200 mg/m^2^/d as the initial dose, which was gradually increased by 50 mg/m^2^/d until the MTD was reached. 5-Fluorouracil was continuously infused for 120 h, and the duration of the chemotherapy cycle was 21 d. Each recruited patient was expected to receive two cycles of induction chemotherapy (IC). Arterial intubation was removed after the completion of concurrent chemoradiotherapy.

### 2.5 Radiotherapy

Radiotherapy by 2-dimensional (2D) techniques or IMRT was planned for all enrolled patients after two cycles of neoadjuvant chemotherapy. Radical radiotherapy was performed at 2.0–2.1 Gy/fraction/d for 5 d per week. The gross volumes of nasopharynx tumours and metastatic neck lymph nodes were contoured using all available diagnostic information sources after two cycles of neoadjuvant chemotherapy. The total dose delivered to the primary tumour was 68–70 Gy/34–35 fractions, and that to the metastatic lymph node sites was 68–70 Gy/34–35 fractions. The patients also received concurrent chemoradiotherapy (CCRT): 40 mg/m^2^ cisplatin intra-arterially every week.

### 2.6 Response and toxicity evaluation criteria

Two weeks after the completion of the second cycle of IC and three months after radiotherapy, treatment responses were estimated by physical examination, nasopharyngoscopy, and nasopharyngeal and neck MRI or CT, according to the Response Evaluation Criteria in Solid Tumours (version 1.1) (11).

Acute toxic effects during IC were classified according to the Common Terminology Criteria for Adverse Events (version 3.0). Acute toxicities occurred during CCRT and three months after radiotherapy. Late adverse reactions were estimated by referring to the Toxicity Criteria of the Radiation Therapy Oncology Group/European Organization for Research and Treatment of Cancer (12). The European Organization for Research and Treatment of Cancer quality of life questionnaire (EORTC QLQ-C30) was used to assess the quality of life (QOL) of patients.

### 2.7 Statistical analyses

The primary endpoints were the MTD of fluorouracil and complete response rate with temporal artery chemotherapy. Secondary endpoints were overall survival (OS), which was defined as the duration between the date of randomisation to the date of death due to any cause, local failure-free survival (LFFS), which was measured from the date of randomisation to the date of local recurrence, regional failure-free survival (RFFS), which was defined from randomisation to regional progression, distant failure-free survival (DFFS), which was the time from the date of randomisation to the date of distant metastasis, progression-free survival (PFS), which was defined from randomization to any treatment failure, and response rates. After the determination of the MTD, a Phase II trial was conducted. The patients in the Phase I portion who were treated with the MTD were entered in the Phase II study. A 2-stage Phase II design was used with a power of 90% and a type I error rate of 5%. A previous study reported that the nasopharyngeal tumours of only 9% of patients with advanced T-stage NPC showed complete response to intravenous induction chemotherapy. We targeted a 33% complete response rate with the temporal artery chemotherapy. Taking into consideration parameters such as dropping out and missing visits, the sample was expanded by 20%, which led to a final sample size of 28 in the Phase II study. After the completion of treatment, all patients were monitored every three months for the first two years, every six months for the following three years, and annually thereafter. Routine clinical follow-up included the following assessments: vital signs, MRI or CT of the nasopharynx and neck, CT scans of the thorax and abdomen, skeletal scintigraphy (performed as clinically indicated), and fiberoptic nasopharyngoscopy. The statistical package for Social Sciences, version 18.0 (SPSS, Chicago, IL, USA) was used for the statistical analysis. A univariate survival analysis was performed, and survival curves were generated using the Kaplan–Meier method.

## 3 Results

All 51 patients had regular follow-ups and physical examinations until death or the latest scheduled assessment. We included all data up to the 17^th^ of August, 2020; the median follow-up time was 162 months for survivors. The baseline demographic and clinical characteristics of the eligible patients are presented in **Table 1**. All patients completed two cycles of IC.

**Table 1:** Baseline characteristics of the study participants.

Characteristics	Median number (range)
Age (y)	18–66 (48)
Sex (n)	
Male	37
Female	14
ECOG score (n)	
0	36
1	15
Histologic feature	
Poorly differentiated squamous carcinoma	45
Well-moderately differentiated squamous carcinoma	6
T stage (n)	
T3	17
T4	34
Clinical stage (n)	
III	13
IVa	32
IVb	6
Follow-up period (m)	162 (13–195)

In the Phase I study, seven dose gradients of 5-fluorouracil were used: 200 (3 cases), 250 (3 cases), 300 (3 cases), 350 (6 cases), 400 (6 cases), 450 (6 cases), and 500 mg/m^2^/d (2 cases). No DLT was observed in the groups administered < 300 mg/m^2^/d of 5-fluorouracil. Only one patient from each group treated with 350, 400, and 450 mg/m^2^/d developed grade III oral mucositis. Therefore, we repeated the corresponding dose in the three patients. No toxic effects were observed in the three patients; hence, the next dose gradient was administered. When the patients received 500 mg/m^2^/d, one patient developed grade III oral mucositis after the completion of the second cycle of IC, and a second patient also experienced DLT (grade III oral mucositis) during the second cycle of chemotherapy; thus, the trial was concluded. The MTD was 450 mg/m^2^/d for 120 h.

**Table 2** shows the various toxic effects experienced by patients in each dose group during IC. A total of 29 patients developed grade 2–3 stomatitis, which was improved by using an oral mucosal repair agent and intravenous nutritional support treatment without affecting the overall treatment schedule. **Table 3** lists the acute and late toxic effects caused by concurrent chemoradiotherapy. Concurrent chemoradiotherapy was temporarily discontinued in four cases (8.0%) because of acute radiation-related toxicities; however, all patients recovered within 5 d (mean, 3 d; range, 2–5 d) after symptomatic treatment. The most common long-term adverse reaction of chemoradiotherapy was grade I or II xerostomia. No late grade ≥ 2 neurological damages, such as brain stem injury, temporal lobe necrosis, and spinal cord injury, were observed. One patient experienced vision loss due to tumour invasion of cranial nerves, which was not related to the induction arterial chemotherapy. None of the patients died owing to toxic effects following therapy or developed infection or thrombosis. Obstruction of ductus arteriosus did not occur in any patient during IC.

**Table 2:** Acute toxic effects during induction chemotherapy and tumour response following induction chemotherapy.

Subtype	Grade	Dose group (mg/m^2^/d)						
		200 (n = 3)	250 (n = 3)	300 (n = 3)	350 (n = 6)	400 (n = 6)	450 (n = 28)	500 (n = 2)
Toxicity								
Leukocytopenia	I	1	0	1	3	2	12	0
	II	0	1	1	1	1	3	1
	III	0	0	0	0	1	1	0
	IV	0	0	0	0	0	0	0
Thrombocytopenia	I	0	1	0	2	1	5	0
	II	0	0	0	0	0	1	0
	III	0	0	0	0	0	0	0
	IV	0	0	0	0	0	0	0
Oral mucositis	I	3	2	3	2	1	11	0
	II	0	1	0	3	4	16	0
	III	0	0	0	1	1	1	2
	IV	0	0	0	0	0	0	0
Vomiting	I	2	1	3	3	5	13	1
	II	1	1	0	2	1	14	1
	III	0	0	0	0	0	0	0
	IV	0	0	0	0	0	0	0
Nausea	I	2	2	3	4	5	34	0
	II	1	1	0	2	1	17	2
	III	0	0	0	0	0	0	0
	IV	0	0	0	0	0	0	0
Diarrhoea	I	0	1	0	1	0	2	0
	II	0	0	0	0	0	0	0
	III	0	0	0	0	0	0	0
	IV	0	0	0	0	0	0	0

**Table 3:** Frequency of acute and late toxicities by type and grade.

Toxicity	Grade 0 (n)	Grade 1 (n)	Grade 2 (n)	Grade 3 (n)	Grade 4 (n)
Acute toxicity					
Neutropenia	11	28	10	2	0
Thrombocytopenia	36	15	0	0	0
Anaemia	8	41	2	0	0
Nausea/vomiting	0	39	12	0	0
Mucositis	0	14	36	1	0
Dermatitis	0	32	19	0	0
Swallowing pain	0	35	15	1	0
Renal-failure	47	4	0	0	0
Late toxicity					
Xerostomia (24th mo)	6	29	16	0	0
Subcutaneous fibrosis	32	19	0	0	0
Trismus	51	0	0	0	0
Hearing impairment	34	16	1	0	0
Cranial nerve palsy	50	1	0	0	0
Vision loss	50	1	0	0	0
Dysphagia	49	2	0	0	0
Temporal lobe necrosis	50	1	0	0	0

After intra-arterial chemotherapy, all 51 (100%) patients achieved T-category down-staging: 35.3% (18/51) reached T0; 39.2% (20/51) reached T1; and 25.5% (13 cases) reached T2 from T3 or T4. There were 23 patients who were canulated on both sides for tumours that crossed the midline. Chemotherapy doses were divided equally on both sides. Of these, 9 had complete remission and 14 had partial remission after induction chemotherapy. None of the patients had progressive disease during IC; complete response was observed in 35.3% of primary tumours and partial response was observed in 64.7%. Furthermore, 43.1% of neck lymph nodes showed complete response and 56.9% showed partial response among patients with lymph node metastasis (**Table 4**). According to the evaluation criteria, both the primary tumour and neck lymph nodes showed a response rate of 100% to chemoradiotherapy.

**Table 4:** Tumour response following induction chemotherapy.

Response	Dose group (mg/m^2^/d)						
	200 (n = 3)	250 (n = 3)	300 (n = 3)	350 (n = 6)	400 (n = 6)	450 (n = 28)	500 (n = 2)
Nasopharyngeal tumour							
CR	0	0	0	1	2	14	1
PR	3	3	3	5	4	14	1
SD	0	0	0	0	0	0	0
PD	0	0	0	0	0	0	0
Regional neck lymph nodes							
CR	0	1	0	2	3	16	0
PR	3	2	3	4	3	12	2
SD	0	0	0	0	0	0	0
PD	0	0	0	0	0	0	0

CR, complete response; PR, partial response; SD, stable disease; PD, progressive disease.

**Figure 1:**
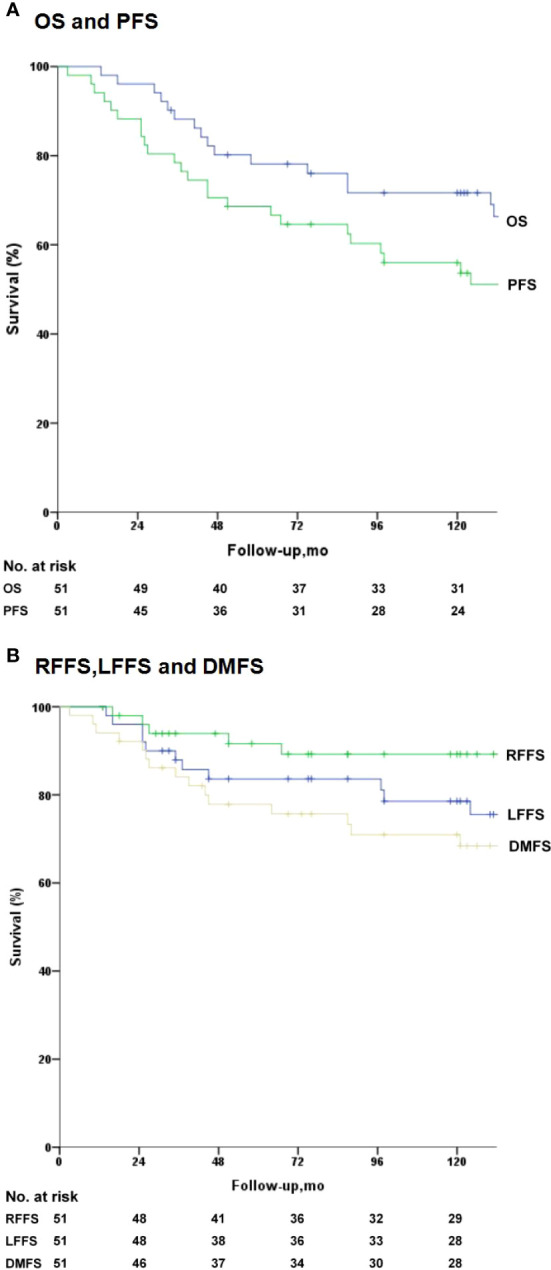
Kaplan–Meier survival analysis for the 51 patients. Median overall survival (OS) was 131 months. **(A)** OS and progression-free survival (PFS). **(B)** Local failure-free survival (LFFS), regional failure-free survival (RFFS), distant failure-free survival (DFFS).

During subsequent follow-ups, 11 patients developed nasopharyngeal failure (8 received 2D radiation, 3 received IMRT), five had neck lymph node recrudescence (3 received 2D radiation, 2 received IMRT), and 15 were confirmed to have distant metastasis *via* imaging (3 exhibited locoregional failure). By the final follow-up, five patients died owing to local failure, 12 owing to distant metastasis, three owing to local failure plus distant metastasis, one owing to a cardiovascular disease, and one owing to a traffic accident. For all patients, the 5- and 10-year survival estimates were: LFFS (83.8% and 78.6%), RFFS (91.6% and 89.2%), DFFS (78.2% and 71.1%), PFS (68.6% and 56.2%), and OS rates (78.0% and 71.7%), respectively (**Figure 1**). Moreover, a positive correlation was affirmed between 5-fluorouracil dose and LC. The optimal dose was 400 mg/m^2^/d for 120 h based on the response, toxicities, and survival.

During the latest follow-up at two years, all patients received a QOL questionnaire. In total, 33 QOL scores, including function, global health status, and symptoms, were calculated as instructed in the EORTC QLQ-C30 scoring manual. The calculated scores are shown in **Table 5**.

**Table 5:** Calculated scores for QLQ-C30 Version 3.0.

Scale name	Mean	Median	Range
Global health status/QOL			
Global health status	77.6	76	34–100
Functional Scales			
Physical functioning	87.7	91	26–100
Role functioning	90.8	94	55–100
Emotional functioning	80.2	81	44–100
Cognitive functioning	85.3	88	56–100
Social functioning	83.7	87	49–98
Symptom scales/items			
Fatigue	25	24	0–78
Nausea and vomiting	5.1	0	0–23
Pain	15.9	14	0–78
Dyspnoea	7.1	0	0–45
Insomnia	22	24	0–89
Appetite loss	6.7	0	0–41
Constipation	15.3	0	0–81
Diarrhoea	5.5	0	0–33
Financial difficulties	24.6	20	0–100

## 4 Discussion

Despite the advances in modern treatment, favourable prognosis is a challenge in patients with T4 NPC (13), particularly in those with intracranial extensions. Although IMRT has advantages, late toxic effects on the central nervous system present a challenge to treatment (14). Su et al. (15) reported that among patients with NPC undergoing treatment with IMRT, those with T1–T2 NPC do not show temporal lobe injury; however, the occurrence rate of T1–T2 NPC is significantly higher in patients with T4 NPC (13.4%) than in those with T3 NPC (3.1%). Adequate dose coverage and adjacent organ protection pose a predicament. Given the chemo-sensitive nature of NPC, IC has potential advantages, including shrinkage of the primary tumour to provide a wider margin for radiotherapy, which is especially beneficial for patients with extensive locoregional infiltration contiguous to critical structures. However, IC does not improve the LC rate as expected. Furthermore, the combination of IC and CCRT has been associated with a significantly lower rate of distant failure than CCRT alone (16). In a retrospective study, 881 patients with locoregionally advanced NPC were analysed, and the results showed that IC perhaps provides the most beneficial effects in patients with bulky or extensive nodal disease (17). Thus, we hypothesised that because IC is always performed intravenously, the tumour tissue does not receive the required drug concentration, and the tumour volume does not reduce significantly. Johnson (18) reported that the nasopharyngeal tumours of only 9% of patients with advanced T-stage NPC show complete response to intravenous neoadjuvant chemotherapy. They suggested that the advanced T-stage is associated with decreased LC and recurrence but not distant metastasis. These results indicate that effective local chemotherapy for patients with advanced T-stage is imperative. The intra-arterial administration of anticancer agents might result in increased levels of anticancer drugs being delivered to tumours, resulting in more potent antitumor effects than intravenous administration (19). In a study on retrograde super-selective intra-arterial chemotherapy and daily concurrent radiotherapy, the OS and LC rates among patients with stages III and IV oral cancer were excellent. At five years, the LC rates in patients with stages III and IV oral cancer were 85.1% and 75.4%, respectively (20).

The PF regimen is one of the recommended protocols for IC in patients with locoregionally advanced NPC; however, the dose of 5-fluorouracil for arterial chemotherapy is still undefined. To the best of our knowledge, this is the first prospective Phase I clinical trial to identify the MTD of 5-fluorouracil administered *via* the superficial temporal artery for 120 h. After two rounds of intra-arterial chemotherapy, the overall response rates (complete plus partial responses) for the primary mass were 100%. All patients achieved T-category down-staging, which resolved the hindrance preventing tumour dose escalation, owing to the anatomical proximity to critical structures in advanced T-stage disease, even in the IMRT era. Consequently, the main late toxicity of radiotherapy in our enrolled patients was xerostomia. No patient developed late neurological dysfunctions, such as brain stem injury, temporal lobe necrosis, and spinal cord damage.

In the present study, grade 3 or 4 toxicities mainly included oral mucositis in five cases during IC. Intra-arterial chemotherapy can deliver a high dose of anticancer drugs to nasopharyngeal tumours, but oral mucositis is a significant toxic effect that can affect swallowing. However, through parenteral nutrition support and symptomatic management, all patients recovered within one week. Newman et al. (21) investigated the swallowing function in patients with head and neck cancer treated with intra-arterial or intravenous CRT and found no significant differences between the groups. These findings indicate that the toxicity of intra-arterial chemotherapy manifests locally and is tolerable.

Au et al. (3) reported the treatment outcomes of NPC after IMRT; the 5-year LFFS, RFFS, DFFS, PFS, and OS for patients with T3 and T4 NPC were 90.3%, 92.5%, 83.5%, 70.3%, 78%, and 76% and 88.8%, 72.7%, 52.1%, and 63.5%, respectively. Heng et al. (22) reported a 5-year survival of 63% in patients with stage T3 NPC and 31% in patients with stage T4 NPC. The poor survival rates might be attributed to the use of conventional radiotherapy. Similarly, disappointing survival rates were observed in the Guangzhou series (23). In our study, the 5-year LFFS, RFFS, DFFS, PFS, and OS rates for the whole group who received 2D radiotherapy were 83.8%, 91.6%,78.2%, 68.6%, and 78.0%, respectively, and the majority of recruited patients were in the T4 stage (66.7%). After prolonged verification, our trial yielded encouraging survival results for patients with advanced T-stage NPC, even in the age of conventional radiotherapy, similar to those of IMRT.

The mean scores of cognitive, physical, role, and social functioning in our group were 85.3, 87.7, 90.8, and 83.7, respectively. Compared with the scores of a trial that included four different radiotherapy techniques (24), the functional scores we obtained were significantly higher than the data of 2D radiotherapy; we also obtained better results in terms of cognitive function than those obtained by IMRT, and locally advanced NPC accounted for only 50.6% in that trial.

Moreover, arterial intubation chemotherapy can facilitate the development of guidelines to treat other locally advanced tumours. A Phase III randomised study evaluating stages II and III breast cancer showed that preoperative chemotherapy is effective in increasing the rate of breast-conserving surgery in patients with locally advanced breast cancer and that locoregional disease-free survival does not decline (25). As reported by Yang et al. (26), reducing the IMRT target volume after IC does not reduce the LC and survival rate in patients with locoregionally advanced NPC, but the doses received by normal tissues decrease and the QOL scores improve. A more effective chemotherapy treatment strategy would shrink the primary tumour significantly, leading to a resectable disease, or would remarkably reduce the high dose volumes required for radiotherapy and maximise the protection of normal tissue. Simultaneously, the LC rate of the tumour would not decrease.

Unlike systemic intravenous chemotherapy, arterial chemotherapy ensures that a high drug concentration is delivered, and organ preservation may be more possible, even in cases of advanced head and neck cancer, avoiding extended surgery and preserving primary organ function. The results of this Phase I/II clinical trial demonstrate that IC is a promising novel treatment strategy for other locally advanced head and neck cancers in combination with the IMRT technique, particularly for advanced T-stage NPC.

To the best of our knowledge, this Phase I/II clinical study is the first to determine the MTD, adverse events, and efficacy of 5-fluorouracil administered continuously for 120 h *via* superficial temporal artery cannulation in patients with advanced T-stage NPC. The limitations of this study are as follows (1): catheter blockage might occur owing to the improper management of the ductus arteriosus, and (2) this method is not suitable for patients with lower cervical lymph node metastasis (3). Although our approach showed good tolerability and safety in our patients, in order to perform super selective temporal artery catheterisation, specially trained operators and confirmation using angiography are required. Furthermore, nurses will need to be extra careful when caring for these patients and report flow interruptions (4). Finally, because it was a phase I/II clinical trial, the sample size was relatively small. A phase III trial is thus needed to demonstrate the value of this approach.

## 5 Conclusion

Continuous pump infusion of 5-fluorouracil for 120 h *via* superficial temporal artery cannulation can lead to significant down-staging in advanced T-stage NPC and result in good tolerability and efficacy, which can lead to better LC with fewer late toxicities of the central nervous system than expected. Our findings warrant validation in Phase II trials using the IMRT technique.

## Data availability statement

The original contributions presented in the study are included in the article/**supplementary material**. Further inquiries can be directed to the corresponding authors.

## Ethics statement

This Phase I/II study was approved (protocol number 200419) by the institutional ethics committees of the Affiliated Hospital of Southwest Medical University. The patients/participants provided their written informed consent to participate in this study.

## Author contributions

LX, formal analysis, validation, project administration, and writing - original draft preparation. YZ, methodology, software, writing – reviewing, and editing. PR, software and data curation. SL, JZ, and QW, investigation and resources. LH, resources and visualization. CS, investigation. JW, conceptualization, supervision, and project administration. All authors contributed to the article and approved the submitted version.

## Funding

The study was funded by the affiliated hospital of Southwest Medical University.

## Acknowledgments

This study has been presented as a digital poster at the 60th ASTRO Annual Meeting. We thank the patients who voluntarily participated in this study and Prof. Liang Shangzhen for his assistance in the catheterisation of the superficial temporal artery (Department of Oral-Maxillofacial Surgery, Affiliated Hospital of Southwest Medical University, Luzhou, China).

## Conflict of interest

The authors declare that the research was conducted in the absence of any commercial or financial relationships that could be construed as a potential conflict of interest.

## Publisher’s note

All claims expressed in this article are solely those of the authors and do not necessarily represent those of their affiliated organizations, or those of the publisher, the editors and the reviewers. Any product that may be evaluated in this article, or claim that may be made by its manufacturer, is not guaranteed or endorsed by the publisher.
