# Supplementary material for: 5-Fluorouracil combined with cisplatin via arterial induction for advanced T-stage nasopharyngeal carcinoma: A 10-year outcome of a phase I/II study
Source: Front Oncol. 2022 Jul 27;12:868070. doi: 10.3389/fonc.2022.868070 (PMC9364084; doi:10.3389/fonc.2022.868070)
Supplement: Supplementary file 1 [file DataSheet_1.docx]

Supplementary Material

# Supplementary Methods

## Superficial temporal artery catheterisation

Before chemotherapy, three-dimensional computed tomography angiography of the carotid artery was performed to identify whether the superficial temporal artery was the main tumour-feeding artery. The entire operation was performed with patients under local anaesthesia by an experienced oral surgeon. An indwelling vascular catheter was retrogradely inserted into the superficial temporal artery. During the operation, methylene blue staining was used to differentiate the skin and mucous membranes from the tumour area, as well as to adjust the depth of the tube and reach the ideal position. The flow in the target artery was checked by computed tomography angiography. If the nasopharyngeal tumour exceeded the midline, bilateral superficial temporal artery cannulas were inserted. Furthermore, pretreatment confirmation of the feeding artery was performed by injecting small amounts of methylene blue.

## Trial design

All anticancer agents were injected using a superficial temporal arterial catheter. Dose-limiting toxicity (DLT) was defined as grade III or above oral mucositis or diarrhoea and grade IV or above other serious toxicities, which were not ameliorated even after 1 week of symptomatic treatment. The Phase I study protocol employed a 3+3 design. In this design, three patients were treated with dose K. If none of the patients experienced DLT, the dose was escalated to K+1. If two or more patients experienced DLT, the dose was de-escalated to K-1. If one patient experienced DLT, three more patients received dose K. If one of the six patients experienced DLT, the dose was escalated to K+1. If two or more of the six patients experienced DLT, the dose was reduced to K-1 and the trial was closed. The dose K-1 was considered to be the maximum tolerated dose. To determine the maximum tolerated dose (MTD) of 5-fluorouracil, we used 200 mg/m^2^/d as the initial dose, which was gradually increased by 50 mg/m^2^/d until the MTD was reached. 5-Fluorouracil was continuously infused for 120 h, and the duration of the chemotherapy cycle was 21 d. Each recruited patient was expected to receive two cycles of induction chemotherapy (IC). Arterial intubation was removed after the completion of concurrent chemoradiotherapy. After the determination of the MTD, a Phase II trial was conducted. The patients in the Phase I portion who were treated with the MTD were entered in the Phase II study. A 2-stage Phase II design was used with a power of 90% and type I error rate of 5%. A previous study reported that the nasopharyngeal tumours of only 9% of patients with advanced T-stage NPC show complete response to intravenous induction chemotherapy. We targeted a 33% complete response rate with the temporal artery chemotherapy. Taking into consideration parameters such as dropping out and missing visits, the sample was expanded by 20%, which led to a final sample size of 28 in the Phase II study.

# Supplementary Data

## The data of MTD

In the Phase I study, seven dose gradients of 5-fluorouracil were used: 200 (3 cases), 250 (3 cases), 300 (3 cases), 350 (6 cases), 400 (6 cases), 450 (6 cases), and 500 mg/m^2^/d (2 cases). No DLT was observed in the groups administered < 300 mg/m^2^/d 5-fluorouracil. Only one patient from each group treated with 350, 400, and 450 mg/m^2^/d developed grade III oral mucositis. Therefore, we repeated the corresponding dose in the three patients. No toxic effects were observed in the three patients; hence, the next dose gradient was administered. When the patients received 500 mg/m^2^/d, one patient developed grade III oral mucositis after the completion of the second cycle of IC, and a second patient also experienced DLT (grade III oral mucositis) during the second cycle of chemotherapy; thus, the trial was concluded. The MTD was 450 mg/m^2^/d for 120 h.
